# Supplementary material for: Tuning moiré excitons and correlated electronic states through layer degree of freedom
Source: Nat Commun. 2022 Aug 16;13:4810. doi: 10.1038/s41467-022-32493-9 (PMC9381773; doi:10.1038/s41467-022-32493-9)
Supplement: Supplementary file 1 — Supplementary Information [file 41467_2022_32493_MOESM1_ESM.pdf]

Supplementary Information: Tuning Moiré Excitons and Correlated Electronic States through Layer Degree of Freedom

Dongxue Chen[1,2#], Zhen Lian[2#], Xiong Huang[3,4#], Ying Su[5#], Mina Rashetnia[3], Li Yan[2], Mark Blei[6], Takashi Taniguchi[7], Kenji Watanabe[8], Sefaattin Tongay[6], Zenghui Wang[1*], Chuanwei Zhang[5*], Yong-Tao Cui[3*], Su-Fei Shi[2,9*]

1. Institute of Fundamental and Frontier Sciences, University of Electronic Science and Technology of China, Chengdu, Sichuan, China
2. Department of Chemical and Biological Engineering, Rensselaer Polytechnic Institute, Troy, NY 12180, USA
3. Department of Physics and Astronomy, University of California, Riverside, California, 92521, USA
4. Department of Materials Science and Engineering, University of California, Riverside, California, 92521, USA
5. Department of Physics, University of Texas at Dallas, Dallas, Texas, 75083, USA
6. School for Engineering of Matter, Transport and Energy, Arizona State University, Tempe, AZ 85287, USA
7. International Center for Materials Nanoarchitectonics, National Institute for Materials Science, 1-1 Namiki, Tsukuba 305-0044, Japan
8. Research Center for Functional Materials, National Institute for Materials Science, 1-1 Namiki, Tsukuba 305-0044, Japan
9. Department of Electrical, Computer & Systems Engineering, Rensselaer Polytechnic Institute, Troy, NY 12180, USA

[#] These authors contributed equally to this work
[*] Corresponding authors: zenghui.wang@uestc.edu.cn, Chuanwei.Zhang@utdallas.edu, yongtao.cui@ucr.edu, shis2@rpi.edu

Table of Contents

Supplementary Note 1: Nature of moiré excitons in 1L/1L WSe_2/WS_2

Supplementary Note 2: Moiré excitons in 2L/1L and 3L/1L WSe_2/WS_2

Supplementary Note 3: Theoretical model of WSe_2/WS_2 heterojunctions

Supplementary Note 4: Sample characterization

Additional Figures: Supplementary Figure 1- Supplementary Figure 11

Additional Table: Supplementary Table 1

Supplementary Note 1：Nature of moiré excitons in 1L/1L WSe_2/WS_2

To reveal the nature of moiré excitons, we first consider the WSe_2 intralayer A exciton described by the effective Hamiltonian

$$H_{\mathrm{A},\boldsymbol{Q}} = \left(E_0 + \frac{\hbar^2\boldsymbol{Q}^2}{2M}\right)\tau_0 + J|\boldsymbol{Q}|\tau_0 + J|\boldsymbol{Q}|\left[\cos(2\theta_{\boldsymbol{Q}})\tau_x + \sin(2\theta_{\boldsymbol{Q}})\tau_y\right]$$

where τ_0 and $\tau_{x,y}$ are the identity matrix and Pauli matrices acting on the valley space [1,2]. $\boldsymbol{Q}$ is the exciton center-of-mass momentum whose polar angle is $\theta_{\boldsymbol{Q}}$ and $M = 0.64m_e$ is the total mass of an electron-hole pair in WSe_2 [3]. The eigenstates and eigenvalues of H_A are:

$$\left|\psi_{\pm,\boldsymbol{Q}}\right\rangle = \frac{1}{\sqrt{2}}\begin{pmatrix} e^{-i\theta_{Q}} \\ \pm e^{i\theta_{Q}} \end{pmatrix}, \qquad E_{\pm,\boldsymbol{Q}} = E_0 + \frac{\hbar^2\boldsymbol{Q}^2}{2M} + J|\boldsymbol{Q}| \pm J|\boldsymbol{Q}|$$

Note that the two eigenstates are degenerate at $\boldsymbol{Q} = \boldsymbol{0}$ and any superposition of $\left|\psi_{\pm,\boldsymbol{0}}\right\rangle$ remains the eigenstate of $H_{A,\boldsymbol{0}}$. Namely, there is an emergent valley pseudospin rotational symmetry, i.e., $H_{A,\boldsymbol{0}}$ is invariant under any pseudospin rotation operation. However, this emergent symmetry is merely induced by combining the two A exciton states from opposite valleys together in $H_{A,\boldsymbol{Q}}$ even when they are not hybridized at $\boldsymbol{Q} = \boldsymbol{0}$. Since valley is a good quantum number for the bright A exciton states, we should fix

$$\left|\psi_{+,\boldsymbol{0}}\right\rangle = \left|X_{A,K}\right\rangle = \begin{pmatrix} 1 \\ 0 \end{pmatrix}, \qquad \left|\psi_{-,\boldsymbol{0}}\right\rangle = \left|X_{A,K'}\right\rangle = \begin{pmatrix} 0 \\ 1 \end{pmatrix}$$

For $\boldsymbol{Q} \neq \boldsymbol{0}$, the nonzero intervalley exchange interaction hybridizes $\left|X_{A,K}\right\rangle$ and $\left|X_{A,K'}\right\rangle$, and breaks the pseudospin rotational symmetry.

In 1L/1L WSe_2/WS_2, the exciton moiré potential reads

$$V_X(\boldsymbol{r}) = \sum_{i=1}^{6} V_i \exp(i\boldsymbol{G}_i \cdot \boldsymbol{r})$$

where $V_{1,3,5} = V\exp(i\phi)$, $V_{2,4,6} = V\exp(-i\phi)$, and $\boldsymbol{G}_{1,3,5} = -\boldsymbol{G}_{2,4,6}$ (see Supplementary Fig. 1a) [4]. $J = 0.04$ eV·nm, $V = 25$ meV and $\phi = 15^{\circ}$ [5]. Then the moiré exciton can be described by the Hamiltonian

$$H_{MX} = H_{A,\boldsymbol{Q}} + V_X(\boldsymbol{r}) \qquad (1)$$

The moiré potential can couple two excitonic states if their momenta are differed by a primitive moiré reciprocal lattice vector $\boldsymbol{G}_i$ with $i \in \{1, \cdots, 6\}$. Therefore, the bright A exciton states $\left|X_{A,K}\right\rangle$ and $\left|X_{A,K'}\right\rangle$ (at γ in Fig. R1a) can couple to the twelve Umklapp states [6] $\{\left|\psi_{\pm,\boldsymbol{G}_i}\right\rangle\}$ in the first shell as

$$\left\langle\psi_{\pm,\boldsymbol{G}_i}\middle|V_X\middle|X_{A,K}\right\rangle = \frac{V_i}{\sqrt{2}}e^{i\theta_{G_i}}, \qquad \left\langle\psi_{\pm,\boldsymbol{G}_i}\middle|V_X\middle|X_{A,K'}\right\rangle = \pm\frac{V_i}{\sqrt{2}}e^{-i\theta_{G_i}}$$

Moreover, the twelve Umklapp states can also mutually couple with their nearest neighbors as

$$\left\langle\psi_{\pm,\boldsymbol{G}_{i+1}}\middle|V_X\middle|\psi_{\pm,\boldsymbol{G}_i}\right\rangle = \frac{V_{i+2}}{2}, \qquad \left\langle\psi_{\pm,\boldsymbol{G}_{i+1}}\middle|V_X\middle|\psi_{\mp,\boldsymbol{G}_i}\right\rangle = \frac{i\sqrt{3}V_{i+2}}{2}$$

where we use the fact that $\boldsymbol{G}_{i+1} - \boldsymbol{G}_i = \boldsymbol{G}_{i+2}$ and $\theta_{\boldsymbol{G}_{i+1}} - \theta_{\boldsymbol{G}_i} = \pi/3$. Here we consider $\boldsymbol{G}_i$ for $i \in \{1, \cdots, 6\}$ forming a loop, i.e., $\boldsymbol{G}_{6+n} = \boldsymbol{G}_n$ and $V_{6+n} = V_n$.

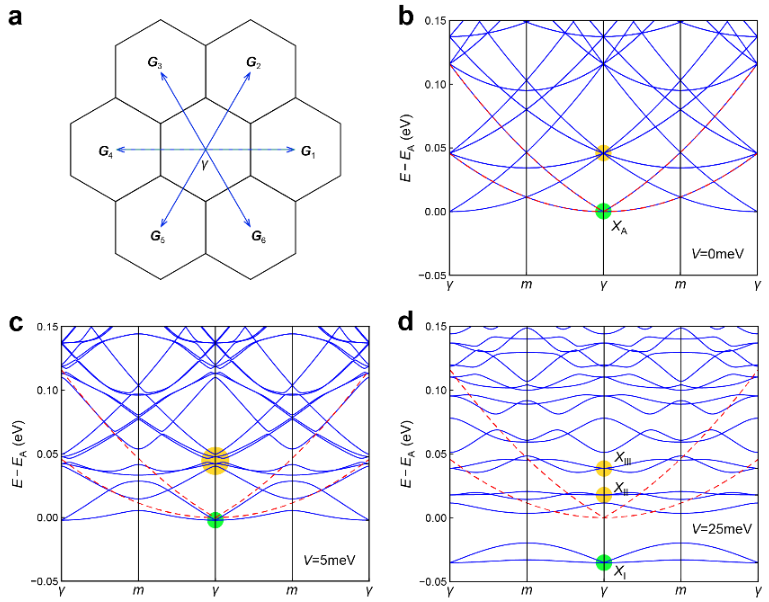

Supplementary Figure 1. (a) Schematic Umklapp scattering induced by the moiré potential. The black hexagons are the MBZs. (b-d) Exciton minibands of 1L/1L WSe_2/WS_2 with $V = 0, 5, 25$ meV. The red dashed curves represent the bare dispersion of WSe_2 intralayer A exciton. The energy bands are plotted along the green dashed path in (a).

Now we treat the moiré potential as a perturbation and consider the leading order corrections to the bright A exciton states as

$$\left|X_{I,+}^{1\mathrm{L}}\right\rangle = \left|X_{A,K}\right\rangle + \sum_{\xi=\pm}\sum_{i=1}^{6} \frac{\langle \psi_{\xi,\boldsymbol{G}_i} | V_X | X_{A,K} \rangle}{E_0 - E_{\xi,\boldsymbol{G}_i}} \left|\psi_{\xi,\boldsymbol{G}_i}\right\rangle$$

$$= \left|X_{A,K}\right\rangle - \sum_{i=1}^{6} \frac{V_i e^{i\theta_{\boldsymbol{G}_i}}}{\sqrt{2}} \left(\frac{\left|\psi_{+,\boldsymbol{G}_i}\right\rangle}{\frac{\hbar^2 b_M^2}{2M} + 2J b_M} + \frac{\left|\psi_{-,\boldsymbol{G}_i}\right\rangle}{\frac{\hbar^2 b_M^2}{2M}} \right) \quad (2)$$

$$\left|X_{I,-}^{1\mathrm{L}}\right\rangle = \left|X_{A,K'}\right\rangle + \sum_{\xi=\pm}\sum_{i=1}^{6} \frac{\langle \psi_{\xi,\boldsymbol{G}_i} | V_X | X_{A,K'} \rangle}{E_0 - E_{\xi,\boldsymbol{G}_i}} \left|\psi_{\xi,\boldsymbol{G}_i}\right\rangle$$

$$= \left|X_{A,K'}\right\rangle - \sum_{i=1}^{6} \frac{V_i e^{-i\theta_{\boldsymbol{G}_i}}}{\sqrt{2}} \left(\frac{\left|\psi_{+,\boldsymbol{G}_i}\right\rangle}{\frac{\hbar^2 b_M^2}{2M} + 2J b_M} - \frac{\left|\psi_{-,\boldsymbol{G}_i}\right\rangle}{\frac{\hbar^2 b_M^2}{2M}} \right) \quad (3)$$

where $b_M = |\boldsymbol{G}_i|$. In principle, the two degenerate bright A exciton states from opposite valleys can hybridize through the second order process mediated by the coupling with $\left|\psi_{\pm,\boldsymbol{G}_i}\right\rangle$. According to the degenerate second order perturbation theory, the effective coupling between $\left|X_{A,K}\right\rangle$ and $\left|X_{A,K'}\right\rangle$ by integrating out the Umklapp states is

$$T_{K,K'} = \sum_{\xi=\pm}\sum_{i=1}^{6} \frac{\langle X_{A,K}|V_X|\psi_{\xi,\mathbf{G}_i}\rangle\langle\psi_{\xi,\mathbf{G}_i}|V_X|X_{A,K'}\rangle}{E_0 - E_{\xi,\mathbf{G}_i}} = \sum_{i=1}^{6}\frac{V^2}{2}e^{-2i\theta_{\mathbf{G}_i}}\left(\frac{2M}{\hbar^2 b_M^2} - \frac{2M}{\hbar^2 b_M^2 + 4Jb_M M}\right) = 0 \quad (4)$$

The effective intervalley coupling vanishes due to the quantum interference since $\sum_{i=1}^{6} e^{-2i\theta_{G_i}} = 0$. Therefore, the two moiré excitons $X_{I,\pm}^{1L}$ inherit components from the Umklapp states and remain degenerate at

$$E_{X_{I,+}^{1L}} = E_0 + \sum_{\xi=\pm}\sum_{i=1}^{6}\frac{\left|\langle\psi_{\xi,\mathbf{G}_i}|V_X|X_{A,K}\rangle\right|^2}{E_0 - E_{\xi,\mathbf{G}_i}} = E_0 - \frac{6V^2M}{\hbar^2 b_M^2 + 4Jb_M M} - \frac{6V^2M}{\hbar^2 b_M^2}$$

$$E_{X_{I,-}^{1L}} = E_0 + \sum_{\xi=\pm}\sum_{i=1}^{6}\frac{\left|\langle\psi_{\xi,\mathbf{G}_i}|V_X|X_{A,K'}\rangle\right|^2}{E_0 - E_{\xi,\mathbf{G}_i}} = E_{X_{I,+}^{1L}}$$

There is no hybridization between $|X_{A,K}\rangle$ and $|X_{A,K'}\rangle$ which would otherwise break the degeneracy.

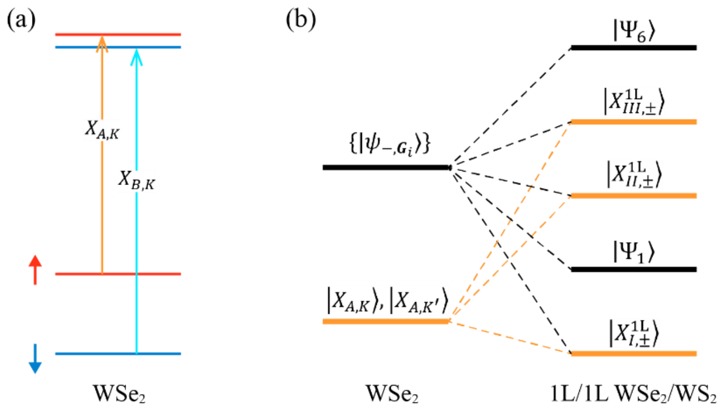

Supplementary Figure 2. (a) Schematic optical interband transitions and the formation of A and B excitons in monolayer WSe_2. The red (blue) energy levels denote the electronic states with spin up (down) at the K valley. (b) The formation of moiré excitons in 1L/1L WSe_2/WS_2 through the hybridization between WSe_2 A exciton states and Umklapp states. The yellow and black energy levels are for the bright and dark excitonic states. The dashed lines denote the hybridization between different excitonic states.

The Umklapp states can also inherit components from the bright A exciton states and generate additional bright moiré excitons (see Supplementary Fig. 2). To show this explicitly, we consider the lowest six degenerate Umklapp states $\{|\psi_{-,\mathbf{G}_i}\rangle\}$. The leading order correction comes from the mutual coupling among themselves. According to the degenerate perturbation theory, diagonalizing the 6×6 matrix whose phenomenological model nonzero elements are

$$\langle\psi_{-,\mathbf{G}_{i+1}}|V_X|\psi_{-,\mathbf{G}_i}\rangle = \frac{V_{i+2}}{2}$$

yields the six perturbed Umklapp states

$$|\psi_1\rangle = \frac{1}{\sqrt{6}}\left(\sum_{i=1,3,6} e^{-\frac{i\phi}{2}}|\psi_{-,\boldsymbol{G}_i}\rangle - \sum_{i=2,4,5} e^{\frac{i\phi}{2}}|\psi_{-,\boldsymbol{G}_i}\rangle\right)$$
$$|\psi_2\rangle = \frac{1}{2}\left(e^{-\frac{i\phi}{2}}|\psi_{-,\boldsymbol{G}_1}\rangle - e^{\frac{i\phi}{2}}|\psi_{-,\boldsymbol{G}_2}\rangle + e^{\frac{i\phi}{2}}|\psi_{-,\boldsymbol{G}_4}\rangle - e^{-\frac{i\phi}{2}}|\psi_{-,\boldsymbol{G}_5}\rangle\right)$$
$$|\psi_3\rangle = \frac{1}{2\sqrt{3}}\left(e^{-\frac{i\phi}{2}}|\psi_{-,\boldsymbol{G}_1}\rangle + e^{\frac{i\phi}{2}}|\psi_{-,\boldsymbol{G}_2}\rangle - 2e^{-\frac{i\phi}{2}}|\psi_{-,\boldsymbol{G}_3}\rangle + e^{\frac{i\phi}{2}}|\psi_{-,\boldsymbol{G}_4}\rangle + e^{-\frac{i\phi}{2}}|\psi_{-,\boldsymbol{G}_5}\rangle - 2e^{\frac{i\phi}{2}}|\psi_{-,\boldsymbol{G}_6}\rangle\right)$$
$$|\psi_4\rangle = \frac{1}{2}\left(e^{-\frac{i\phi}{2}}|\psi_{-,\boldsymbol{G}_1}\rangle + e^{\frac{i\phi}{2}}|\psi_{-,\boldsymbol{G}_2}\rangle - e^{\frac{i\phi}{2}}|\psi_{-,\boldsymbol{G}_4}\rangle - e^{-\frac{i\phi}{2}}|\psi_{-,\boldsymbol{G}_5}\rangle\right)$$
$$|\psi_5\rangle = \frac{1}{2\sqrt{3}}\left(e^{-\frac{i\phi}{2}}|\psi_{-,\boldsymbol{G}_1}\rangle - e^{\frac{i\phi}{2}}|\psi_{-,\boldsymbol{G}_2}\rangle - 2e^{-\frac{i\phi}{2}}|\psi_{-,\boldsymbol{G}_3}\rangle - e^{\frac{i\phi}{2}}|\psi_{-,\boldsymbol{G}_4}\rangle + e^{-\frac{i\phi}{2}}|\psi_{-,\boldsymbol{G}_5}\rangle + 2e^{\frac{i\phi}{2}}|\psi_{-,\boldsymbol{G}_6}\rangle\right)$$
$$|\psi_6\rangle = \frac{1}{\sqrt{6}}\left(\sum_{i=1,3,6} e^{-\frac{i\phi}{2}}|\psi_{-,\boldsymbol{G}_i}\rangle + \sum_{i=2,4,5} e^{\frac{i\phi}{2}}|\psi_{-,\boldsymbol{G}_i}\rangle\right)$$

Here $|\psi_1\rangle$ and $|\psi_6\rangle$ are nondegenerate with $E_1 = E_0 + \frac{\hbar^2 b_M^2}{2m} - V$ and $E_6 = E_0 + \frac{\hbar^2 b_M^2}{2m} + V$, while $|\psi_{2,3}\rangle$ and $|\psi_{4,5}\rangle$ are both double degenerate with $E_{2,3} = E_0 + \frac{\hbar^2 b_M^2}{2M} - \frac{V}{2}$ and $E_{4,5} = E_0 + \frac{\hbar^2 b_M^2}{2M} + \frac{V}{2}$. Furthermore, we consider the first-order correction to $\{|\psi_i\rangle\}$ from their coupling with $|X_{A,K}\rangle$ and $|X_{A,K'}\rangle$ as

$$|\Psi_i\rangle = |\psi_i\rangle + \sum_{\tau=K,K'} \frac{\langle X_{A,\tau}|V_X|\psi_i\rangle}{E_i - E_0}|X_{A,\tau}\rangle$$

that yields

$$|\Psi_1\rangle = |\psi_1\rangle$$
$$|\Psi_2\rangle = |\psi_2\rangle - \frac{(\sqrt{3}+3i)V\sin\frac{3\phi}{2}}{2\sqrt{2}\left(\frac{\hbar^2 b_M^2}{2M} - \frac{V}{2}\right)}|X_{A,K}\rangle - \frac{(\sqrt{3}-3i)V\sin\frac{3\phi}{2}}{2\sqrt{2}\left(\frac{\hbar^2 b_M^2}{2M} - \frac{V}{2}\right)}|X_{A,K'}\rangle \quad (5)$$
$$|\Psi_3\rangle = |\psi_3\rangle + \frac{(3-\sqrt{3}i)V\sin\frac{3\phi}{2}}{2\sqrt{2}\left(\frac{\hbar^2 b_M^2}{2M} - \frac{V}{2}\right)}|X_{A,K}\rangle + \frac{(3+\sqrt{3}i)V\sin\frac{3\phi}{2}}{2\sqrt{2}\left(\frac{\hbar^2 b_M^2}{2M} - \frac{V}{2}\right)}|X_{A,K'}\rangle \quad (6)$$
$$|\Psi_4\rangle = |\psi_4\rangle + \frac{(3-\sqrt{3}i)V\cos\frac{3\phi}{2}}{2\sqrt{2}\left(\frac{\hbar^2 b_M^2}{2M} + \frac{V}{2}\right)}|X_{A,K}\rangle - \frac{(3+\sqrt{3}i)V\cos\frac{3\phi}{2}}{2\sqrt{2}\left(\frac{\hbar^2 b_M^2}{2M} + \frac{V}{2}\right)}|X_{A,K'}\rangle \quad (7)$$
$$|\Psi_5\rangle = |\psi_5\rangle + \frac{(\sqrt{3}+3i)V\cos\frac{3\phi}{2}}{2\sqrt{2}\left(\frac{\hbar^2 b_M^2}{2M} + \frac{V}{2}\right)}|X_{A,K}\rangle - \frac{(\sqrt{3}-3i)V\cos\frac{3\phi}{2}}{2\sqrt{2}\left(\frac{\hbar^2 b_M^2}{2M} + \frac{V}{2}\right)}|X_{A,K'}\rangle \quad (8)$$
$$|\Psi_6\rangle = |\psi_6\rangle$$

Because $|\Psi_{2,3}\rangle$ and $|\Psi_{4,5}\rangle$ inherit components from the bright A excitons, they become the bright moiré excitons as

$$|X_{II,+}^{1\mathrm{L}}\rangle = |\Psi_2\rangle, \quad |X_{II,-}^{1\mathrm{L}}\rangle = |\Psi_3\rangle, \quad |X_{III,+}^{1\mathrm{L}}\rangle = |\Psi_4\rangle, \quad |X_{III,-}^{1\mathrm{L}}\rangle = |\Psi_5\rangle \quad (9)$$

where $\left|X_{II,\pm}^{1L}\right\rangle$ and $\left|X_{III,\pm}^{1L}\right\rangle$ are both double degenerate as

$$E_{X_{II,+}^{1L}} = E_2 + \sum_{\tau=K,K'} \frac{\left|\langle X_{A,\tau}|V_X|X_{II,+}^{1L}\rangle\right|^2}{E_2 - E_0} = E_0 + \frac{\hbar^2 b_M^2}{2M} - \frac{V}{2} + \frac{3V^2 \sin^2\frac{3\phi}{2}}{\frac{\hbar^2 b_M^2}{2M} - \frac{V}{2}}$$

$$E_{X_{II,-}^{1L}} = E_3 + \sum_{\tau=K,K'} \frac{\left|\langle X_{A,\tau}|V_X|X_{II,-}^{1L}\rangle\right|^2}{E_3 - E_0} = E_{X_{II,+}^{1L}}$$

$$E_{X_{III,+}^{1L}} = E_4 + \sum_{\tau=K,K'} \frac{\left|\langle X_{A,\tau}|V_X|X_{III,+}^{1L}\rangle\right|^2}{E_4 - E_0} = E_0 + \frac{\hbar^2 b_M^2}{2M} + \frac{V}{2} + \frac{3V^2 \cos^2\frac{3\phi}{2}}{\frac{\hbar^2 b_M^2}{2M} + \frac{V}{2}}$$

$$E_{X_{III,-}^{1L}} = E_5 + \sum_{\tau=K,K'} \frac{\left|\langle X_{A,\tau}|V_X|X_{III,-}^{1L}\rangle\right|^2}{E_5 - E_0} = E_{X_{III,+}^{1L}}$$

Therefore, any superposition of $\left|X_{II,\pm}^{1L}\right\rangle$ or $\left|X_{III,\pm}^{1L}\right\rangle$ are still the bright moiré exciton states. The nondegenerate $|\Psi_1\rangle$ and $|\Psi_6\rangle$ are orthogonal to the bright A exciton states and remain dark (see Supplementary Fig. 2b). These results are consistent with our numerical simulation of Eq. (1) by the plane wave expansion method, as shown in Supplementary Fig.1b-d. There is another contribution to the first order correction of $\{|\psi_i\rangle\}$ from their coupling with $\{\psi_{+,\boldsymbol{G}_i}\}$, which does not affect the brightness of the moiré excitons and is omitted for simplification.

Supplementary Note 2：Moiré excitons in 2L/1L and 3L/1L WSe_2/WS_2

Hybrid excitons in 2L WSe_2

To understand the moiré excitons in 2L/1L WSe_2/WS_2, we first consider the interlayer hybridization in 2L WSe_2. Due to the AB-stacking, the 1st WSe_2 layer is rotated by 180° with respect to the 2nd WSe_2 layer. Therefore, the energy bands of the two layers have opposite spin polarization in the same valley. At the K and K' points, the spin-conserved interlayer tunneling is forbidden for conduction band electrons due to the symmetry constrain, while it is allowed for valence band holes (see Supplementary Fig. 3a) [7]. The hybridization between two valence bands (with same spin) in different layers can be described by the Hamiltonian $\begin{pmatrix} \lambda & t_\perp \\ t_\perp & -\lambda \end{pmatrix}$ where $2\lambda = 0.456$ eV is the valence band splitting in 1L WSe_2 and $t_\perp = 0.067$ eV is the interlayer hopping [7]. The interlayer hybridization enlarges the valence band splitting to $2\sqrt{\lambda^2 + t_\perp^2}$ and redistributes the valence band holes whose wave function has a small portion of $\sim \frac{t_\perp}{t_\perp + 2\lambda} = 12.8\%$ in the other layer.

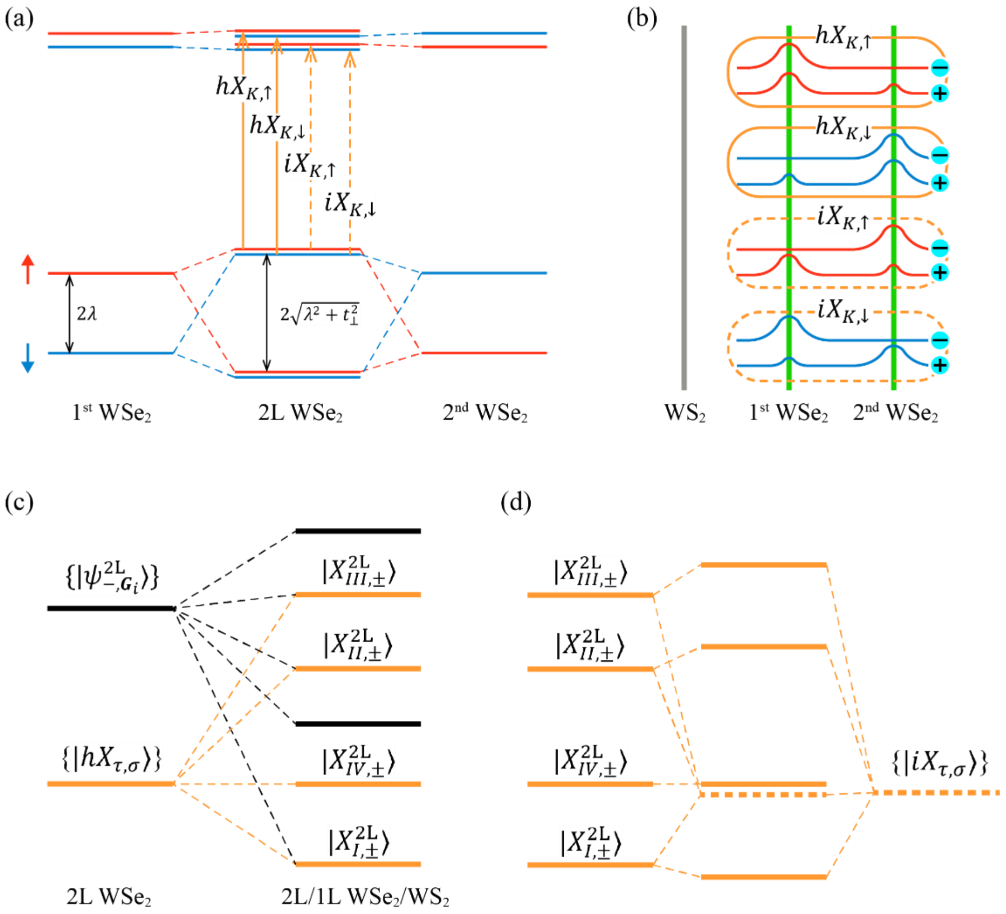

Supplementary Figure 3. (a) Schematic optical interband transitions and the formation of hybrid excitons at the K valley of 2L WSe_2. (b) The spatial charge distribution of intralayer-like and interlayer-like hybrid excitons in 2L WSe_2. (c) The formation of moiré excitons in 2L/1L WSe_2/WS_2 through the hybridization between 2L WSe_2 intralayer-like exciton states and Umklapp states. (d) The energy shifts of moiré excitons through their hybridization with 2L WSe_2 interlayer-like exciton states in 2L/1L WSe_2/WS_2. The yellow and black solid energy levels are for the bright and dark excitonic states. The yellow dashed energy levels denote the intralayer-like exciton states with very weak oscillator strength. The dashed lines indicate the hybridization between different excitonic states.

The hybrid valence bands enable four possible optical interband transitions in the K valley, as shown in Supplementary Fig. 3a, and their time-reversal counterparts in the $K^{'}$ valley [8]. Here the predominate interband transitions (marked by yellow solid arrows) lead to the degenerate intralayer-like hybrid excitons $hX_{K,\uparrow}$ and $hX_{K,\downarrow}$, while the subordinate interband transitions (marked by yellow dashed arrows) generate the degenerate interlayer-like hybrid excitons $iX_{K,\uparrow}$ and $iX_{K,\downarrow}$, as shown in Supplementary Fig. 3b. The hybrid excitons $hX_{K^{'},\sigma}$ and $iX_{K^{'},\sigma}$ (where the spin index $\sigma = \uparrow, \downarrow$) in the $K^{'}$ valley can be obtained by the time-reversal symmetry. The oscillator strength of interlayer-like excitons $iX_{\tau,\sigma}$ (where the valley index $\tau = K, K^{'}$) is much weaker than that of intralayer-like excitons $hX_{\tau,\sigma}$. The energy difference between $hX_{\tau,\sigma}$ and $iX_{\tau,\sigma}$ depends on the conduction band splitting (see Supplementary Fig. 3a) which is about 37 meV in WSe_2 [4].

Because the binding energy of $iX_{\tau,\sigma}$ is smaller than that of $hX_{\tau,\sigma}$ due to the larger electron-hole separation, the energy difference between $hX_{\tau,\sigma}$ and $iX_{\tau,\sigma}$ is smaller than the conduction band splitting and cannot be resolved in the experiment (see Supplementary Fig. 4). It naturally explains why the resonant peak in 1L WSe_2 is sharper than that in 2L WSe_2. Furthermore, there is a redshift of the resonant peak in 2L WSe_2 compared with that in 1L WSe_2 by about 30 meV. This could be due to the change of dielectric environment as well as the reduction of energy gap in 2L WSe_2 (see Supplementary Fig. 3a). Therefore, the hybrid excitons $hX_{\tau,\sigma}$ and $iX_{\tau,\sigma}$ in 2L WSe_2 have lower energy than the intralayer A excitons $X_{A,\tau}$ in 1L WSe_2.

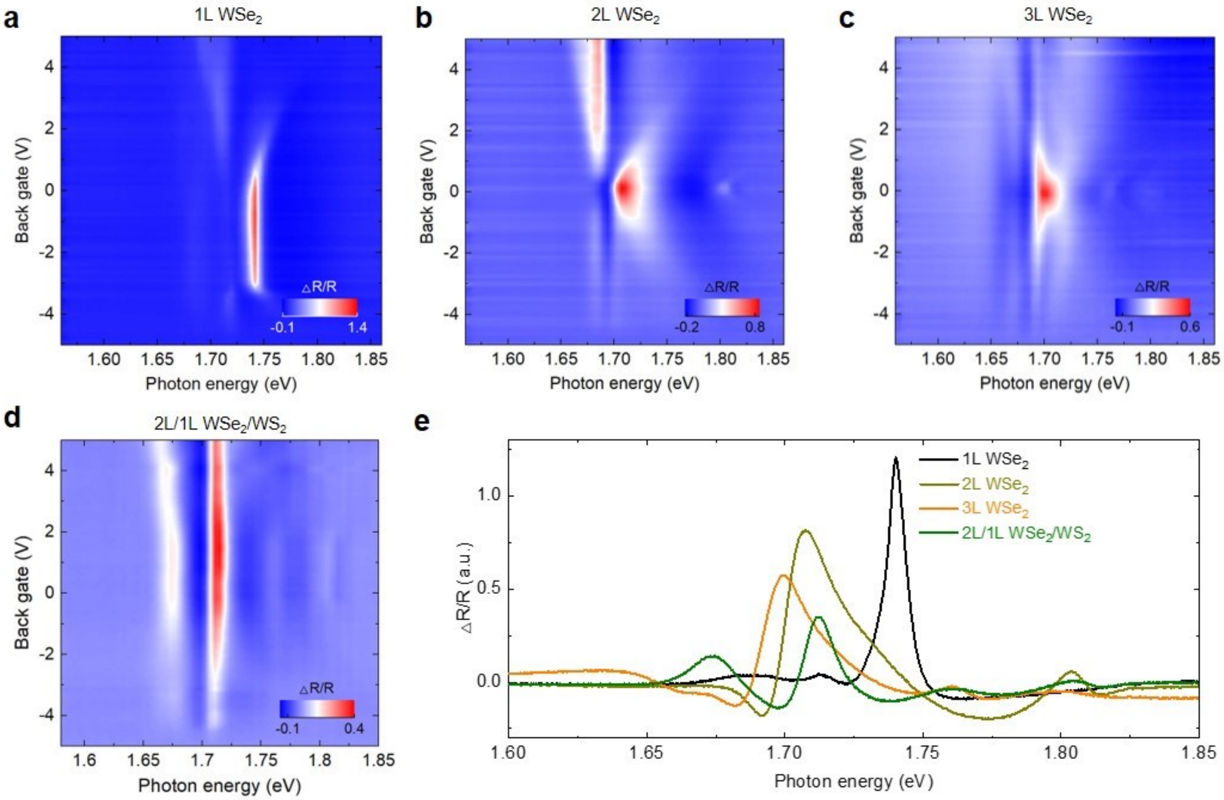

Supplementary Figure 4. (a-d) Differential reflectance spectra as a function of the gate voltage at the region of 1L, 2L, 3L WSe_2 and 2L/1L WSe_2/WS_2. (e) Differential reflectance spectra of different regions at zero gate voltage. All data were from device D3, taken at 5 K.

Possible mechanism for moiré excitons in 2L/1L and 3L/1L WSe_2/WS_2

Now we consider the moiré excitons in 2L/1L WSe_2/WS_2. For the predominant intralayer-like hybrid excitons $\{hX_{\tau,\sigma}\}$, the moiré potential will affect $hX_{K,\uparrow}$ and $hX_{K',\downarrow}$ which are mainly distributed in the 1st WSe_2 layer interfacing WS_2, while $hX_{K,\downarrow}$ and $hX_{K',\uparrow}$ highly localized in the 2nd WSe_2 layer are expected to be unaffected (see Supplementary Fig. 3b). According to the same mechanism in 1L/1L WSe_2/WS_2, the moiré potential will hybridize $|hX_{K,\uparrow}\rangle$ and $|hX_{K',\downarrow}\rangle$ with the Umklapp states $\{|\psi^{2L}_{\xi,\boldsymbol{G}_i}\rangle\}$ and lead to three double degenerate moiré excitons $|X^{2L}_{\mu,\pm}\rangle$ (where $\mu = I, II, III$) through the Umklapp scattering in 2L/1L WSe_2/WS_2 (see Supplementary Fig. 3c). The

moiré exciton states $\left|X_{\mu,\pm}^{2L}\right\rangle$ should be in the similar form as $\left|X_{\mu,\pm}^{1L}\right\rangle$ in Eq. (2,3,5-9) but with the 1L WSe_2 $\left|X_{A,K}\right\rangle$, $\left|X_{A,K^{'}}\right\rangle$, $\left|\psi_{\xi,\boldsymbol{G}_i}\right\rangle$ replaced by the 2L WSe_2 $\left|hX_{K,\uparrow}\right\rangle$, $\left|hX_{K^{'},\downarrow}\right\rangle$, $\left|\psi_{\xi,\boldsymbol{G}_i}^{2L}\right\rangle$. The remaining $\left|hX_{K,\downarrow}\right\rangle$ and $\left|hX_{K^{'},\uparrow}\right\rangle$ in the 2nd WSe_2 layer give rise to the $\left|X_{IV,\pm}^{2L}\right\rangle$ whose resonant energy is coincident with that in 2L WSe_2 (see Supplementary Fig. 4). Therefore, the four resonant peaks observed in 2L/1L WSe_2/WS_2 can be essentially explained by considering the intralayer-like hybrid excitons in 2L WSe_2 coupled to the moiré potential.

Another important feature of the moiré excitons observed in 2L/1L WSe_2/WS_2 is that there is a redshift in $X_{I,\pm}^{2L}$ but blueshifts in $X_{II,\pm}^{2L}$ and $X_{III,\pm}^{2L}$ compared with those in 1L/1L WSe_2/WS_2 (see Fig. 1b of the main text). To understand the energy shifts of moiré excitons, we propose a possible mechanism by further considering the interlayer-like hybrid excitons $\{iX_{\tau,\sigma}\}$. It is noted that $hX_{\tau,\sigma}$ and $iX_{\tau,\sigma}$ [with $(\tau,\sigma)=(K,\uparrow)$ or $(K^{'},\downarrow)$] share the same valence band holes mainly in the 1st WSe_2 layer but have different conduction band electrons in the 1st and 2nd WSe_2 layers, respectively (see Supplementary Fig. 3b). Although the interlayer tunneling between conduction bands is hindered by the symmetry at K and $K^{'}$ points [7], it can be nonzero away from these high-symmetry points. Therefore, the Umklapp states $\left\{\left|\psi_{\xi,\boldsymbol{G}_i}^{2L}\right\rangle\right\}$ in 2L WSe_2 should be formed by electrons and holes (with finite momenta away from K and $K^{'}$) in the hybrid conduction and valence bands, respectively. Meanwhile, the interlayer-like excitons $iX_{\tau,\sigma}$ is about half distributed in the 1st WSe_2 layer and should also feel a moiré potential $V_X^{'}(\boldsymbol{r})$ that leads to the coupling with $\left|\psi_{\xi,\boldsymbol{G}_i}^{2L}\right\rangle$. This paves the way for the hybridization between $\left|X_{\mu,\pm}^{2L}\right\rangle$ and $\left|iX_{\tau,\sigma}\right\rangle$ with the coupling constant $t_\mu=\left\langle iX_{\tau,\sigma}\left|V_X^{'}(\boldsymbol{r})\right|X_{\mu,\pm}^{2L}\right\rangle$ since $\left|X_{\mu,\pm}^{2L}\right\rangle$ contain the components of $\left|\psi_{\xi,\boldsymbol{G}_i}^{2L}\right\rangle$. Furthermore, the Umklapp components dominate in $\left|X_{II,\pm}^{2L}\right\rangle$ and $\left|X_{III,\pm}^{2L}\right\rangle$ that can result in stronger hybridization with $\left|iX_{\tau,\sigma}\right\rangle$. Then this hybridization is expected to induce the energy shifts of moiré excitons, as sketched in Fig. R3d. Note that $\left|X_{IV,\pm}^{2L}\right\rangle$ highly localized in the second WSe_2 layer is barely affected by the hybridization. The similar analysis can be extended to 3L/1L WSe_2/WS_2 in which additional hybrid excitons can be induced by the interlayer tunneling between valence bands in the 2nd and 3rd WSe_2 layers. In this case, the additional hybrid excitons away from the WSe_2/WS_2 interface do not affect the moiré excitons. Therefore, the moiré excitons $X_{\mu,\pm}^{3L}$ in 3L/1L WSe_2/WS_2 are nearly identical to $X_{\mu,\pm}^{2L}$ in 2L/1L WSe_2/WS_2, as shown in Fig. 1b of the main text.

To evaluate $t_\mu=\left\langle iX_{\tau,\sigma}\left|V_X^{'}(\boldsymbol{r})\right|X_{\mu,\pm}^{nL}\right\rangle$ (where $n=2$ or 3), one needs to know the exact hybrid exciton states in nL WSe_2 and the moiré potentials for different hybrid excitons in nL/1L WSe_2/WS_2, which to our knowledge remains unknow and is beyond the scope of this work. Further DFT studies could be stimulated by our experimental results. Nevertheless, we expect the essential physics associated with the energy shifts of moiré excitons can be captured by the 4×4 model Hamiltonian

$$H=\begin{pmatrix} E_{iX} & t_I & t_{II} & t_{III} \\ t_I^* & E_I & 0 & 0 \\ t_{II}^* & 0 & E_{II} & 0 \\ t_{III}^* & 0 & 0 & E_{III} \end{pmatrix} \qquad (10)$$

in the basis of $\{|iX\rangle, |X_I^{nL}\rangle, |X_{II}^{nL}\rangle, |X_{III}^{nL}\rangle\}$. Here we omit the subscript for simplification because each moiré exciton is double degenerate due to the time-reversal symmetry. Because the interlayer-like hybrid exciton iX has very weak oscillator strength and cannot be resolved in the experiment, we approximately treat it as a dark exciton. Diagonalizing the model Hamiltonian Eq. (10) yields the hybrid moiré excitons with enlarged energy separation. The magnitude of t_μ can be estimated from the energy shifts of moiré exciton resonances. To show the optical response of the moiré system, we calculate the real part of the optical conductivity that gives the optical absorption [3]. According to the experimental data, we take $E_I = 1.662$ eV, $E_{II} = 1.715$ eV, and $E_{III} = 1.753$ eV. We approximate the energy of iX by $E_{iX} = 1.700$ eV since it cannot be resolved in the experiment. $t_I = -0.03$ eV, $t_{II} = -0.04$ eV, and $t_{III} = 0.04i$ eV are chosen to match the experimental results in Fig. 1b. The numerical results are shown in Fig. 2c-e for 1L/1L, 2L/1L, and 3L/1L WSe_2/WS_2, respectively. Here we also consider the bright hybrid excitons highly in the upper WSe_2 layers that give rise to $X_{IV}^{2L/3L}$. In particular, the two sub-resonances around $E = 1.69$ eV in Fig. 2e are consistent with the broad resonant peak X_{IV}^{3L} in Fig. 1b which splits into the two sub-resonances in the p-doped site in Fig. 1e.

Supplementary Note 3：Theoretical model of WSe_2/WS_2 heterojunctions

The low-energy states of a monolayer transitional metal dichalcogenide (TMDC) can be described by the effective Dirac Hamiltonian [9]

$$h_{\boldsymbol{k},\tau} = at(\tau k_x\sigma_x + k_y\sigma_y) + \frac{\Delta}{2}\sigma_z - \lambda\tau\frac{\sigma_z - 1}{2}s_z$$

where a is the lattice constant, t is the intralayer hopping, Δ is the energy gap, and λ is the intrinsic spin-orbit coupling (SOC). $\tau = \pm 1$ is the valley index, $\sigma_{x,y,z}$ are the Pauli matrices for two basis orbitals $\left\{|d_{z^2}\rangle, \frac{1}{\sqrt{2}}\left(|d_{x^2-y^2}\rangle + i\tau|d_{xy}\rangle\right)\right\}$, and $s_{x,y,z}$ are the Pauli matrices for spin. The 1L/1L WSe_2/WS_2 heterojunction has the type-II band alignment and the valence band maximum is from WSe_2 that can be described by the continuum model

$$H_{\boldsymbol{k},\tau}^{1L} = h_{\boldsymbol{k},\tau} + V(\boldsymbol{r}), \qquad V(\boldsymbol{r}) = 2V_0\sum_{i=1}^{3}\cos(\boldsymbol{G}_i\cdot\boldsymbol{r} + \phi) \tag{11}$$

Where the low-energy states of WSe_2 couples to the moiré potential $V(\boldsymbol{r})$ [10,11]. Here $\boldsymbol{G}_i = \frac{4\pi}{\sqrt{3}L_M}\left(\cos\frac{2\pi i}{3}, \sin\frac{2\pi i}{3}\right)$ are the moiré reciprocal lattice vectors and the moiré superlattice constant $L_M = a/\sqrt{\delta^2 + \theta^2}$ is determined by the twist angle θ and lattice mismatch $\delta = (a - a')/a' = 4\%$ where a (a') is the lattice constant of WSe_2 (WS_2). According to the first-principal calculation, the model parameters are $a = 3.31$ Å, $a' = 3.19$ Å, $t = 1.19$ eV, $\Delta = 1.60$ eV, $\lambda = 0.23$ eV, $V_0 = 15$

meV, and $\phi = \pi/4$ [8,9,11]. Here we focus on the angle-aligned WSe_2/WS_2 heterojunctions with $\theta = 0°$.

It is straightforward to generalize the continuum model of 1L/1L WSe_2/WS_2 to 2L/1L WSe_2/WS_2

$$H_{\boldsymbol{k},\tau}^{2\mathrm{L}} = \begin{pmatrix} h_{-\boldsymbol{k},-\tau} & t_\perp \dfrac{1-\sigma_z}{2} \\ t_\perp \dfrac{1-\sigma_z}{2} & h_{\boldsymbol{k},\tau} + V(\boldsymbol{r}) \end{pmatrix} \tag{12}$$

and 3L/1L WSe_2/WS_2

$$H_{\boldsymbol{k},\tau}^{3\mathrm{L}} = \begin{pmatrix} h_{\boldsymbol{k},\tau} & t_\perp \frac{1-\sigma_z}{2} & 0 \\ t_\perp \frac{1-\sigma_z}{2} & h_{-\boldsymbol{k},-\tau} & t_\perp \frac{1-\sigma_z}{2} \\ 0 & t_\perp \frac{1-\sigma_z}{2} & h_{\boldsymbol{k},\tau} + V(\boldsymbol{r}) \end{pmatrix} \tag{13}$$

where $t_\perp = 0.067$ eV is the interlayer hopping between $\frac{1}{\sqrt{2}}(|d_{x^2-y^2}\rangle \pm i|d_{xy}\rangle)$ orbitals of two adjacent WSe_2 layers [7]. In AB-stacked multilayer WSe_2, the second layer is rotated by 180° with respect to the first layer and third layer, and it acquires a opposite valley index $-\tau$ and momentum $-\boldsymbol{k}$ in Eq. (12) and (13). By using the plane wave expansion, the continuum models Eq. (11), (12) and (13) yield the valence moiré minibands in Fig. 3a-c for 1L/1L, 2L/1L, and 3L/1L WSe_2/WS_2, respectively. The bandwidth of the top valence miniband in the three heterojunctions is shown in Supplementary Fig. 5.

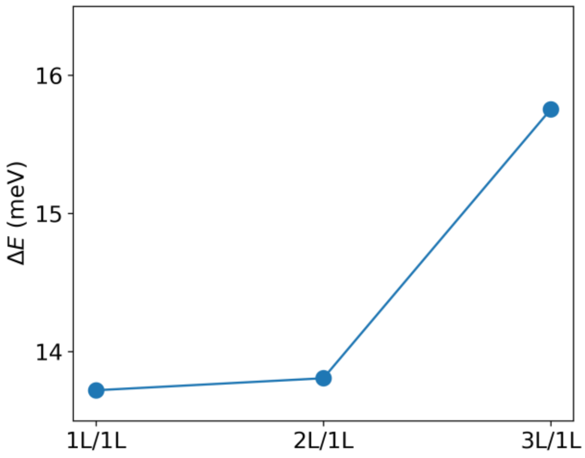

Supplementary Figure 5. The bandwidth of the top valence miniband in 1L/1L, 2L/1L, and 3L/1L WSe_2/WS_2.

Supplementary Note 4：Sample characterization

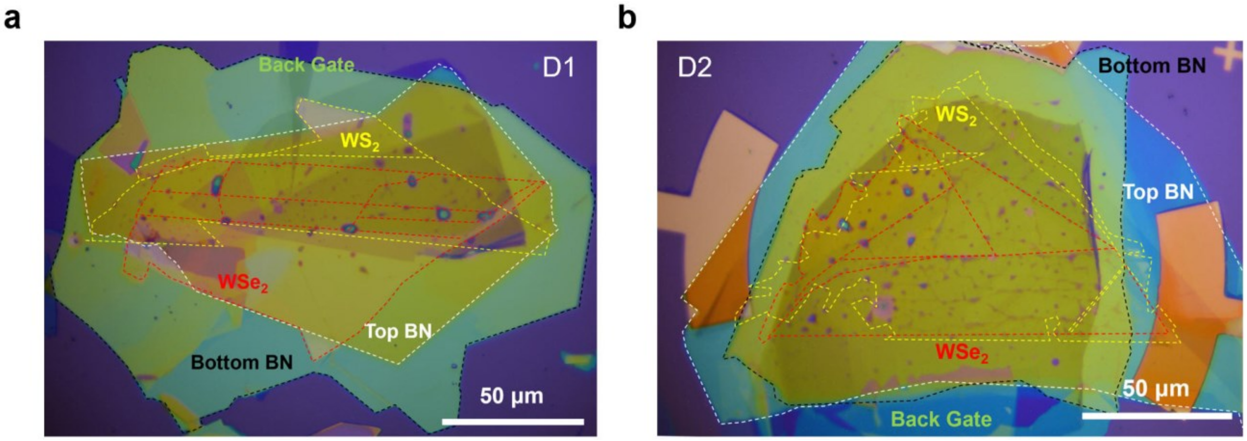

Supplementary Figure 6. Optical image of a back-gate WSe_2/WS_2 device （D1） used in the (a) main text and (b) Supplementary Information (D2).

The optical reflectance from a thin flake of 2D materials is related to its absorption [12,13] and thus a powerful method to determine the thickness of the 2D materials such as WSe_2. The microscope images of three WSe_2 flakes (including a standard WSe_2 flake, device D1, and device D2) are shown in Supplementary Fig. 7a-c. We can apply optical contrast analysis method [12,13] to realize thickness identification by numerically analyzing the RGB optical images of the samples.

The reflectance contrast (R contrast, defined as $(R_{substrate} – R_{sample})/R_{substrate}$) for these three pieces of WSe_2 flake is also calculated (the red channel), and the values for different layer regions are plotted in Supplementary Fig. 7d. It is evident that WSe_2 regions with a certain layer number share similar R contrast, while the increase of one single layer will increase the R contrast by one “step”. Thus, by comparing with the R contrasts of standard WSe_2 flake and the WSe_2 flakes used in D1 and D2, we can accurately confirm the number of layers of WSe_2.

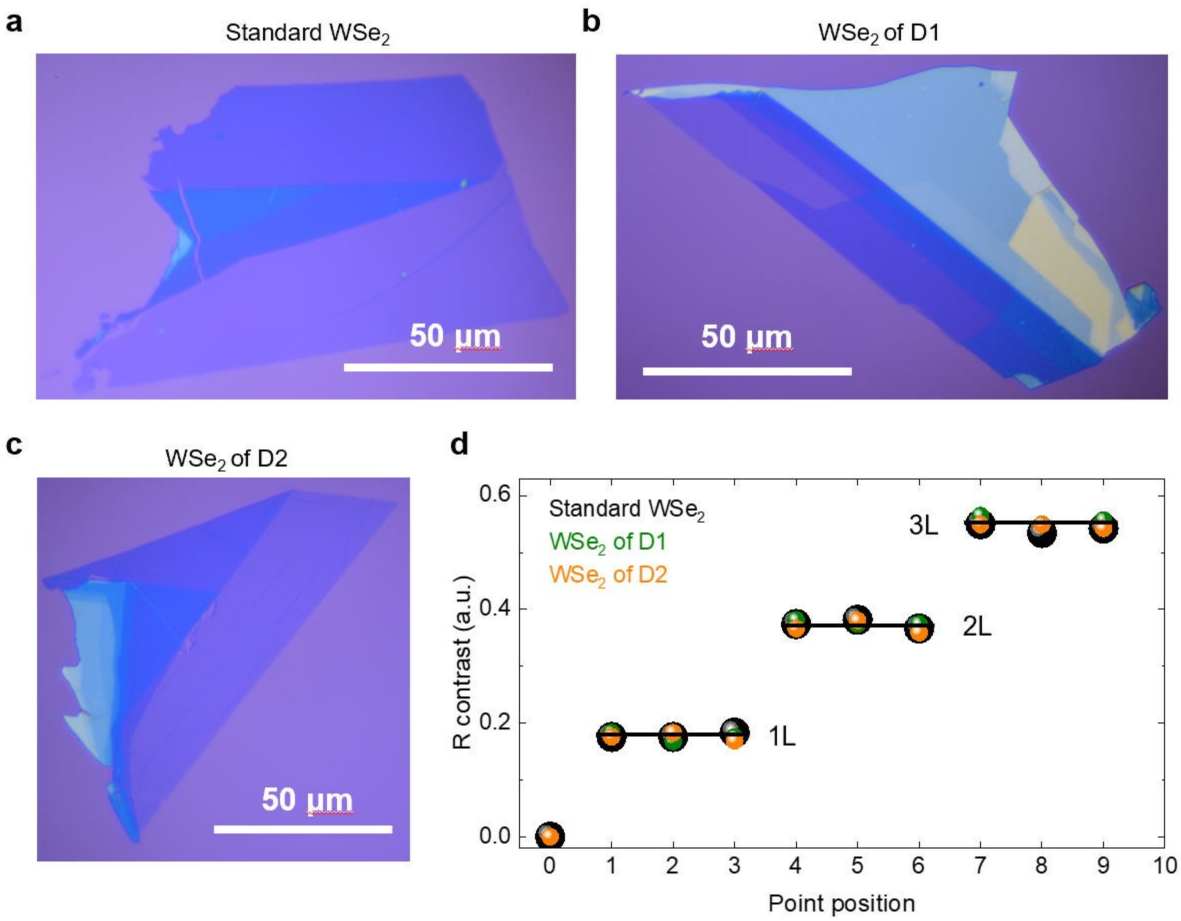

Supplementary Figure 7. Optical images of (a) standard WSe_2, (b) WSe_2 prepared for D1and (c) WSe_2 prepared for D2. (d) Reflectance contrast of the standard WSe_2 sample, the WSe_2 flake for D1 and D2.

We further confirm the layer thickness through AFM measurements (Supplementary Fig. 8). For the standard WSe_2 sample, we overlayer the AFM topography measurements with the microscope image. It is evident that each layer adds to about 0.74 ± 0.03 nm in height, consistent with previous reports [14].

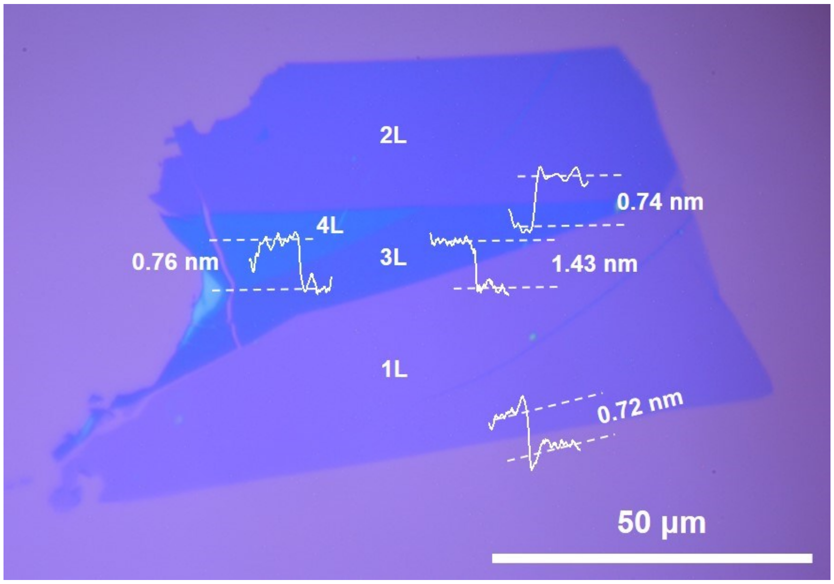

Supplementary Figure 8. AFM measurement results of the standard WSe_2 flake.

Finally, the layer assignment is consistent with the Raman measurements. Supplementary Fig. 9 shows the Raman spectra taken from the different regions on the standard sample using 532 nm laser excitation. A Raman peak is observed at 308.0 cm^{-1} on the 2L WSe_2 region and at 307.7 cm^{-1} on the 3L WSe_2 region, yet it is absent on the monolayer WSe_2 region, as shown in Supplementary Fig. 9c. We also observe a splitting between the A_{1g} mode and the E_{2g}^1 mode on the 2L WSe_2 region and the 3L WSe_2 region, shown in Supplementary Fig. 9a. All these observations are consistent with the Raman spectra reported in Ref. [15].

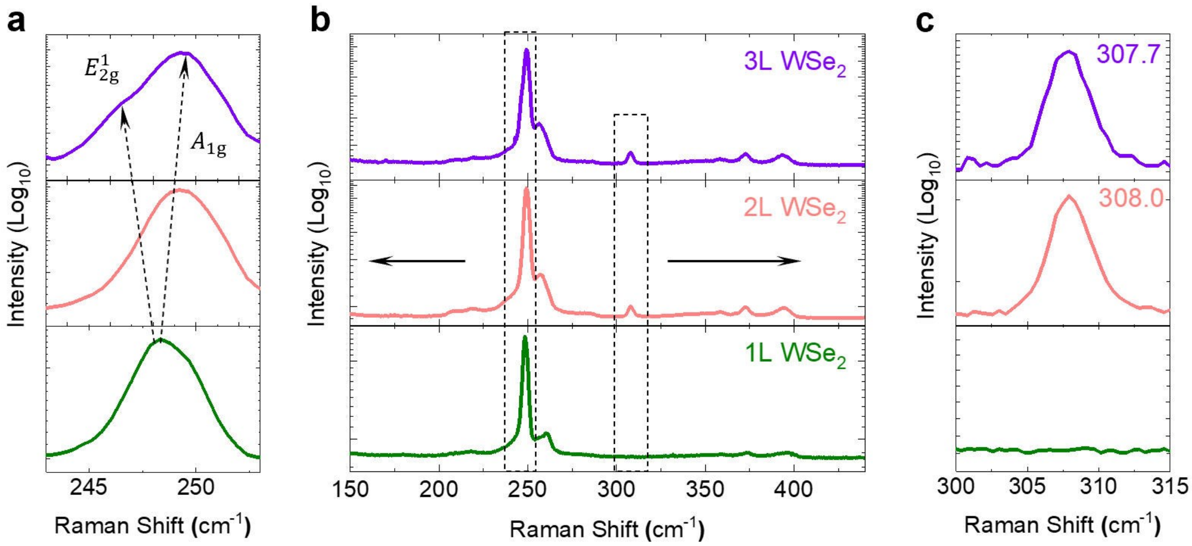

Supplementary Figure 9. Raman spectra of 1L, 2L and 3L WSe_2 from the standard sample. （a） Zoomed in Raman spectra of left black dashed box in (b). (c) Zoomed in Raman spectra of right black dashed box in (b).

Supplementary Note 5：Optical spectra characterization of devices

Device D2 is similar to the device D1 (data presented in the main text) and has well-defined 1L/1L, 2L/1L and 3L/1L WSe_2/WS_2 regions, with the optical image shown in Supplementary Fig. 6b. The optical spectra from D2 are similar to what we presented in the main text, which we include in the Supplementary Fig. 10, which show consistent results compared with the device shown in the main text (D1, Fig. 1).

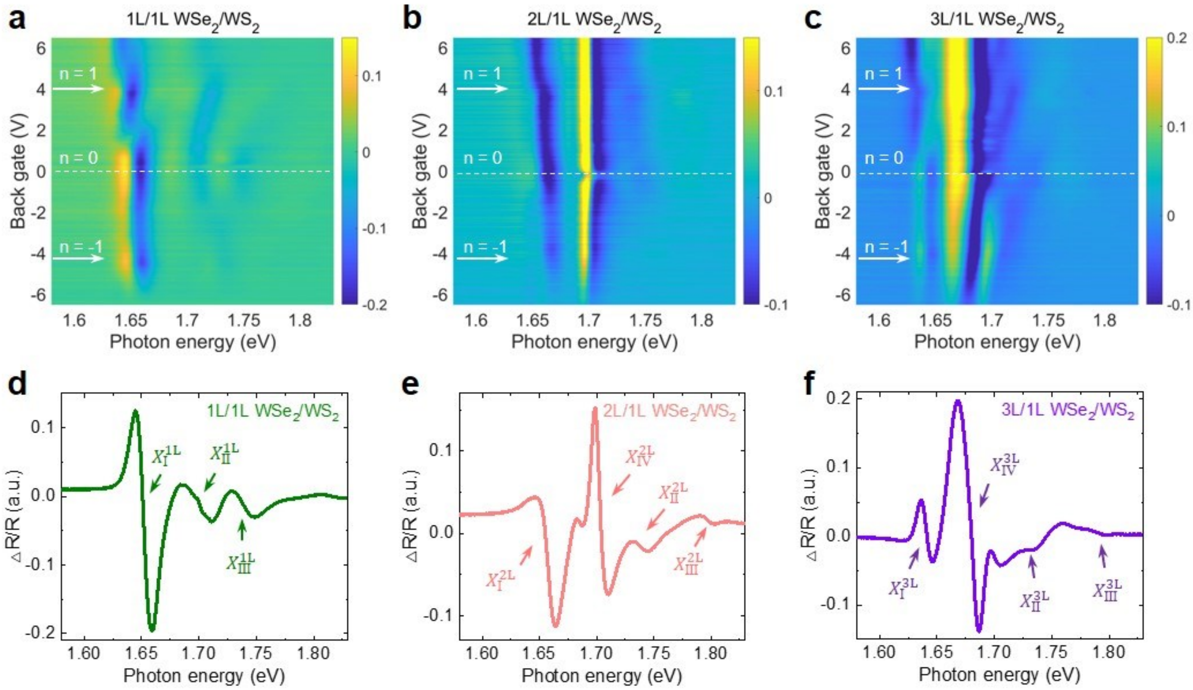

Supplementary Figure 10. (a), (b) and (c) are the differential reflectance spectra as a function of the gate voltage (density of carriers) at the region of 1L/1L, 2L/1L, and 3L/1L WSe_2/WS_2. All data were taken at 10 K. (d), (e) and (f) Differential reflectance spectra of different regions at zero gate voltage.

In total, we have studied the moiré excitons of 9 different samples, and all the data are consistent with the one presented in the main text. We have summarized the results in Supplementary Table 1.

Supplementary Table 1. Exciton resonances from different samples.

Device	Region	X_I (eV)	X_{II} (eV)	X_{III} (eV)	X_{IV} (eV)	Device	Region	X_I (eV)	X_{II} (eV)	X_{III} (eV)	X_{IV} (eV)
D1	1L/1L WSe_2/WS_2	1.662	1.715	1.753		D5	2L/1L WSe_2/WS_2	1.657	1.742	1.794	1.705
	2L/1L WSe_2/WS_2	1.642	1.728	1.793	1.693	D6	1L/1L WSe_2/WS_2	1.687	1.737	1.772	
	3L/1L WSe_2/WS_2	1.645	1.730	1.785	1.677		2L/1L WSe_2/WS_2	1.665	1.757	1.797	1.701
D2	1L/1L WSe_2/WS_2	1.652	1.699	1.739		D7	1L/1L WS_2/WSe_2	1.687	1.725	1.765	
	2L/1L WSe_2/WS_2	1.657	1.737	1.797	1.703		1L/2L WS_2/WSe_2	1.674	1.747	1.799	1.710
	3L/1L WSe_2/WS_2	1.642	1.728	1.789	1.678	D8	1L/1L WSe_2/WS_2	1.685	1.723	1.763	
D3	2L/1L WSe_2/WS_2	1.683	1.772	1.811	1.712		2L/1L WSe_2/WS_2	1.666	1.753	1.795	1.706
	3L/1L WSe_2/WS_2	1.669	1.756	1.824	1.717	D9	1L/1L WSe_2/WS_2	1.687	1.748	1.792	
D4	2L/1L WSe_2/WS_2	1.661	1.755	1.801	1.697		2L/1L WSe_2/WS_2	1.652	1.754	1.803	1.704

Gate-dependent PL spectra of the interlayer excitons for the device shown in the main text (device D1) in Supplementary Fig. 11. The data from the 1L/1L WSe_2/WS_2 clearly show the emergence of the correlated insulating states at fractional fillings (see Supplementary Fig. 11a). The n=1 (-1) is the Mott insulator state for the half filling corresponding to one electron (hole) per moiré superlattice. The fillings at 1/3, 2/3, 1/4 and 1/2 corresponding to correlated insulating states we reported earlier [16,17], and the capability of revealing them directly in the PL spectra demonstrates the high quality of the data. The PL of interlayer excitons are weaker in 2L/1L and 3L/1L WSe_2/WS_2 regions, as shown in Supplementary Fig. 11b, c.

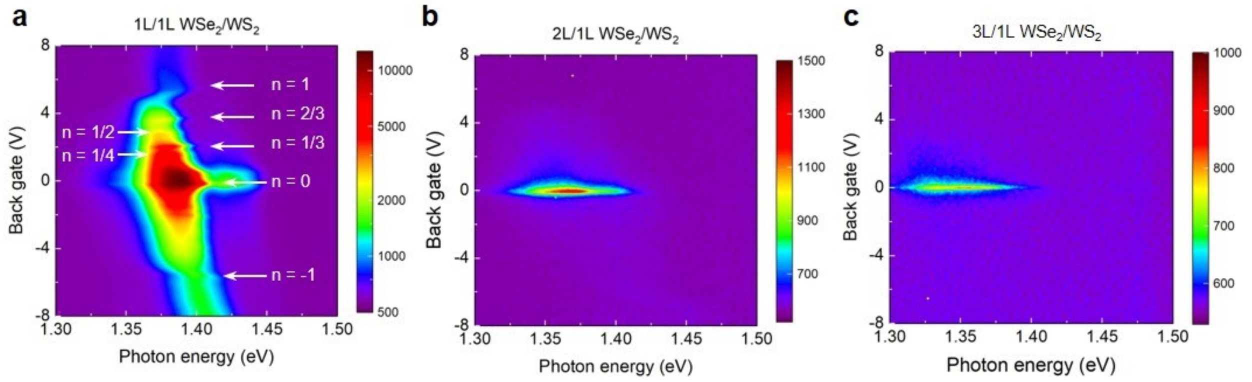

Supplementary Figure 11. PL spectra from (a) 1L/1L, (b) 2L/1L and (c) 3L/1L region of D1. All data were taken at 4.5 K.

Supplementary References

1. Yu, H., Liu, G.-B., Gong, P., Xu, X. & Yao, W. Dirac cones and Dirac saddle points of bright excitons in monolayer transition metal dichalcogenides. *Nature Commun.* **5**, 3876 (2014).

2. Wu, F., Qu, F. & MacDonald, A. H. Exciton band structure of monolayer MoS_2. *Phys. Rev. B* **91**, 075310 (2015).

3. Kormányos, A. et al. k·p theory for two-dimensional transition metal dichalcogenide semiconductors. *2D Mater.* **2**, 022001 (2015).

4. Wu, F., Lovorn, T. & MacDonald, A. H. Topological exciton bands in moiré heterojunctions. *Phys. Rev. Lett.* **118**, 147401 (2017).

5. Jin, C. H. et al. Observation of moire excitons in WSe_2/WS_2 heterostructure superlattices. *Nature* **567**, 76–80 (2019).

6. Shimazaki, Y. et al. Optical signatures of periodic charge distribution in a Mott-like correlated insulator state. *Phys. Rev. X* **11**, 021027 (2021).

7. Gong, Z. et al. Magnetoelectric effects and valley-controlled spin quantum gates in transition metal dichalcogenide bilayers. *Nature Commun.* **4**, 15 (2013).

8. Hsu, W. et al. Tailoring excitonic states of van der Waals bilayers through stacking configuration, band alignment, and valley spin. Sci. Adv. 5, eaax7407 (2019).

9. Xiao, D., Liu, G.-B., Feng, W., Xu, X. & Yao, W. Coupled spin and valley physics in monolayers of MoS_2 and other group-VI dichalcogenides. *Phys. Rev. Lett.* **108**, 196802 (2012).

10. Wu, F., Lovorn, T., Tutuc, E. & MacDonald, A. H. Hubbard model physics in transition metal dichalcogenide moiré bands. *Phys. Rev. Lett.* **121**, 026402 (2018).

11. Zhang, Y., Yuan, N. F. Q. & Fu, L. Moire quantum chemistry: charge transfer in transition metal dichalcogenide superlattices. *Phys. Rev. B*. **102**, 201115 (2020).

12. Bing D. et al. Optical contrast for identifying the thickness of two-dimensional materials. *Opt. Commun*. **406**, 128-138 (2018).

13. Taghavi N. S. et al. Thickness determination of MoS_2, $MoSe_2$, WS_2 and WSe_2 on transparent stamps used for deterministic transfer of 2D materials. *Nano Res*. **12**, 1691–1695 (2019).

14. Han A. et al. Growth of 2H stacked WSe_2 bilayers on sapphire. *Nanoscale Horiz*. **4**, 1434-1442 (2019).

15. Zhao W. et al. Lattice dynamics in mono- and few-layer sheets of WS_2 and WSe_2. *Nanoscale*. 5, 9677-9683 (2013).

16. Huang X. et al. Correlated insulating states at fractional fillings of the WS_2/WSe_2 moiré lattice. *Nat. Phys*. **17**, 715–719 (2021).

17. Miao S. et al. Strong interaction between interlayer excitons and correlated electrons in WSe_2/WS_2 moiré superlattice. *Nat. Commun*. **12**, 3608 (2021).
